# *N*-(4,6-Dimeth­oxy­pyrimidin-2-yl)-2-(3-methyl­phen­yl)acetamide

**DOI:** 10.1107/S1600536811054493

**Published:** 2011-12-23

**Authors:** A. S. Praveen, Jerry P. Jasinski, James A. Golen, H. S. Yathirajan, B. Narayana

**Affiliations:** a Department of Studies in Chemistry, University of Mysore, Manasagangotri, Mysore 570 006, India; b Department of Chemistry, Keene State College, 229 Main Street, Keene, NH 03435-2001, USA; c Department of Studies in Chemistry, Mangalore University, Mangalagangotri, 574 199, India

## Abstract

In the title compound, C_15_H_17_N_3_O_3_, the dihedral angle between the pyrimidine and benzene rings is 87.0 (7)°. In the crystal, mol­ecules are linked into inversion dimers with *R*_2_^2^(8) graph-set motifs by a pair of N—H⋯O hydrogen bonds. Weak C—H⋯O hydrogen bonds and inter­molecular π–π inter­actions [centroid–centroid distance = 3.544 (1) Å] are also observed.

## Related literature

For the pyrimidine ring in vitamins, see: Cox (1968 ▶). For barbitone, the first barbiturate hypnotic sedative, see: Russell (1945 ▶). For the similarity of related *N*-substituted 2-aryl­acetamides to the lateral chain of natural benzyl­penicillin, see: Mijin & Marinkovic (2006 ▶); Mijin *et al.* (2008 ▶). For the coordination abilities of amides, see: Wu *et al.* (2008 ▶, 2010 ▶). For related structures, see: John *et al.* (2010 ▶); Nogueira *et al.* (2010 ▶); Praveen *et al.* (2011 ▶); Selig *et al.* (2010 ▶); Wen *et al.* (2010 ▶). For standard bond lengths, see: Allen *et al.* (1987 ▶).
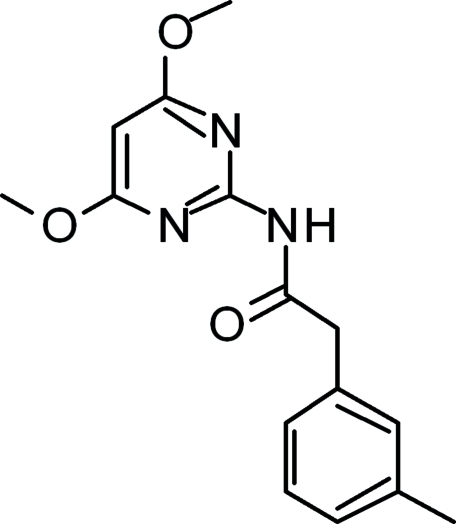


## Experimental

### 

#### Crystal data

C_15_H_17_N_3_O_3_
*M* *_r_* = 287.32
Triclinic, 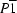

*a* = 7.1536 (6) Å
*b* = 8.2070 (7) Å
*c* = 13.8259 (10) Å
α = 74.420 (7)°
β = 86.540 (6)°
γ = 69.186 (8)°
*V* = 730.30 (10) Å^3^
*Z* = 2
Mo *K*α radiation
μ = 0.09 mm^−1^
*T* = 173 K
0.42 × 0.34 × 0.22 mm

#### Data collection

Oxford Diffraction Xcalibur Eos Gemini diffractometer
Absorption correction: multi-scan (*CrysAlis RED*; Oxford Diffraction, 2010 ▶) *T*_min_ = 0.961, *T*_max_ = 0.980
8350 measured reflections
4735 independent reflections
3887 reflections with *I* > 2σ(*I*)
*R*_int_ = 0.020

#### Refinement

*R*[*F* ^2^ > 2σ(*F* ^2^)] = 0.047
*wR*(*F* ^2^) = 0.132
*S* = 1.02
4735 reflections
197 parameters
H atoms treated by a mixture of independent and constrained refinement
Δρ_max_ = 0.37 e Å^−3^
Δρ_min_ = −0.22 e Å^−3^

### 

Data collection: *CrysAlis PRO* (Oxford Diffraction, 2010 ▶); cell refinement: *CrysAlis PRO*; data reduction: *CrysAlis RED* (Oxford Diffraction, 2010 ▶); program(s) used to solve structure: *SHELXS97* (Sheldrick, 2008 ▶); program(s) used to refine structure: *SHELXL97* (Sheldrick, 2008 ▶); molecular graphics: *SHELXTL* (Sheldrick, 2008 ▶); software used to prepare material for publication: *SHELXTL*.

## Supplementary Material

Crystal structure: contains datablock(s) global, I. DOI: 10.1107/S1600536811054493/is5034sup1.cif

Structure factors: contains datablock(s) I. DOI: 10.1107/S1600536811054493/is5034Isup2.hkl

Supplementary material file. DOI: 10.1107/S1600536811054493/is5034Isup3.cml

Additional supplementary materials:  crystallographic information; 3D view; checkCIF report

## Figures and Tables

**Table 1:** Hydrogen-bond geometry (Å, °)

*D*—H⋯*A*	*D*—H	H⋯*A*	*D*⋯*A*	*D*—H⋯*A*
N3—H3*N*⋯O3^i^	0.875 (15)	1.979 (15)	2.8535 (12)	176.0 (14)
C3—H3⋯O2^ii^	0.93	2.52	3.4459 (12)	177

Symmetry codes: (i) 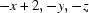; (ii) 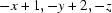.

**Fig. 1.**
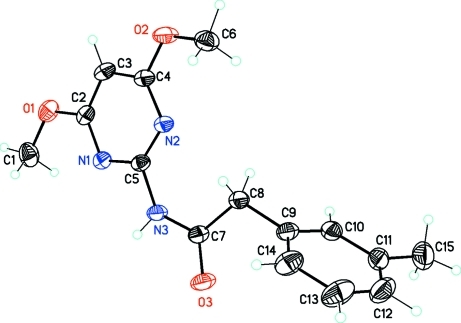
Molecular structure of the title compound, showing the atom labeling scheme and 50% probability displacement ellipsoids.

**Fig. 2.**
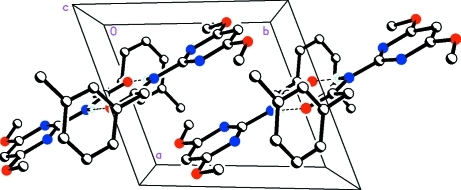
Packing diagram of the title compound viewed along the c axis. Dashed line represent N—H···O hydrogen bonds forming an R22(8) graph-set motif. The remaining H atoms have been removed for clarity.

## supplementary crystallographic information

### Comment

The pyrimidine ring is found in vitamins like thiamine, riboflavin and folic acid (Cox, 1968).  Barbitone, the first barbiturate hypnotic sedative and anticonvulsant, is a pyrimidine derivative (Russell, 1945). N-Substituted 2-arylacetamides are very interesting compounds because of their structural similarity to the lateral chain of natural benzylpenicillin (Mijin *et al.*, 2006, 2008). Amides are also used as ligands due to their excellent coordination abilities (Wu *et al.*, 2008, 2010).

Crystal structures of some acetamidederivatives, *viz*., 2-[(5,7-dibromoquinolin-8-yl)oxy]-N-(2-methoxyphenyl)acetamide (Wen *et al.*, 2010), N-(4-bromophenyl)-2-(2-thienyl)acetamide (Nogueira *et al.*, 2010), N-[4-(benzylsulfamoyl)phenyl]acetamide (John *et al.*, 2010), 2-(4-fluorophenyl)-N-{4-[6-(4-fluorophenyl)-2,3-dihydroimidazo [2,1-b][1,3]thiazol-5-yl]pyridin-2-yl}acetamide (Selig *et al.*, 2010) and recently from our laboratories, N-(3-chloro-4-fluorophenyl)-2-(naphthalen-1-yl)acetamide (Praveen *et al.*, 2011) have been reported. As part of our ongoing studies of amides, the title compound is synthesized and its crystal structure is reported.

In the crystal structure of the title compound, C_15_H_17_N_3_O_3_, the dihedral angle between the pyrimidine and benzene rings is 93.0 (7)° (Fig. 1). Bond lengths are in normal ranges (Allen *et al.*, 1987).  Crystal packing is stabilized by N—H···O hydrogen bonds forming an R^2^_2_(8) graph-set motif (Fig. 2). Weak C—H···O (Table 1) and π—π intermolecular interactions [centroid-centroid distance = 3.544 (1) Å] are also observed.

### Experimental

To a stirred solution of (3-methylphenyl)acetic acid (1 g, 6.65 mmol), triethylamine (1.34 g, 13.31 mmol) and 4,6-dimethoxypyrimidin-2-amine (1.02 g, 6.65 mmol) in dichloromethane (10 ml), 1-(3-dimethylaminopropyl)-3-ethylcarbodiimide HCl (1.52 g, 7.93 mmol) was added at 273 K. Reaction mixture was stirred at room temperature for 3 h. After the completion of the reaction, the reaction mixture was poured to ice cold water and the layers were separated. Organic layer was washed with 10% aq.NaHCO_3_ solution (10 ml), brine (10 ml), dried over anhydrous Na_2_SO_4_, filtered and concentrated under vacuum to obtain the crude product which was triturated with ethanol and filtered to afford 1.62 g of the title compound (I) as a white solid in 84% yield. Single crystals were grown from ethanol by the slow evaporation method (m.p. 381-382 K).

### Refinement

Atom H3N was located in a difference Fourier map and refined isotropically.  All of the remaining H atoms were placed in their calculated positions and then refined using the riding model with C—H = 0.93 Å (CH), 0.97 Å (CH_2_) or 0.96 Å (CH_3_). The *U*_iso_(H) values were set to 1.2 (CH, CH_2_) or 1.5 CH_3_) times *U*_eq_ of the parent atom.

### Crystal data

**Table:**

C_15_H_17_N_3_O_3_	*Z* = 2
*M**_r_* = 287.32	*F*(000) = 304
Triclinic, *P*1	*D*_x_ = 1.306 Mg m^−^^3^
Hall symbol: -P 1	Mo *K*α radiation, λ = 0.71073 Å
*a* = 7.1536 (6) Å	Cell parameters from 3427 reflections
*b* = 8.2070 (7) Å	θ = 3.1–32.3°
*c* = 13.8259 (10) Å	µ = 0.09 mm^−^^1^
α = 74.420 (7)°	*T* = 173 K
β = 86.540 (6)°	Chunk, colorless
γ = 69.186 (8)°	0.42 × 0.34 × 0.22 mm
*V* = 730.30 (10) Å^3^	

### Data collection

**Table:**

Oxford Diffraction Xcalibur Eos Gemini diffractometer	4735 independent reflections
Radiation source: Enhance (Mo) X-ray Source	3887 reflections with *I* > 2σ(*I*)
graphite	*R*_int_ = 0.020
Detector resolution: 16.1500 pixels mm^-1^	θ_max_ = 32.3°, θ_min_ = 3.1°
ω scans	*h* = −10→10
Absorption correction: multi-scan (*CrysAlis RED*; Oxford Diffraction, 2010)	*k* = −10→12
*T*_min_ = 0.961, *T*_max_ = 0.980	*l* = −20→20
8350 measured reflections	

### Refinement

**Table:**

Refinement on *F*^2^	Primary atom site location: structure-invariant direct methods
Least-squares matrix: full	Secondary atom site location: difference Fourier map
*R*[*F*^2^ > 2σ(*F*^2^)] = 0.047	Hydrogen site location: inferred from neighbouring sites
*wR*(*F*^2^) = 0.132	H atoms treated by a mixture of independent and constrained refinement
*S* = 1.02	*w* = 1/[σ^2^(*F*_o_^2^) + (0.0664*P*)^2^ + 0.156*P*] where *P* = (*F*_o_^2^ + 2*F*_c_^2^)/3
4735 reflections	(Δ/σ)_max_ < 0.001
197 parameters	Δρ_max_ = 0.37 e Å^−^^3^
0 restraints	Δρ_min_ = −0.22 e Å^−^^3^

### Special details

**Table:**

Geometry. All esds (except the esd in the dihedral angle between two l.s. planes) are estimated using the full covariance matrix. The cell esds are taken into account individually in the estimation of esds in distances, angles and torsion angles; correlations between esds in cell parameters are only used when they are defined by crystal symmetry. An approximate (isotropic) treatment of cell esds is used for estimating esds involving l.s. planes.
Refinement. Refinement of *F*^2^ against ALL reflections. The weighted *R*-factor *wR* and goodness of fit *S* are based on *F*^2^, conventional *R*-factors *R* are based on *F*, with *F* set to zero for negative *F*^2^. The threshold expression of *F*^2^ > σ(*F*^2^) is used only for calculating *R*-factors(gt) *etc*. and is not relevant to the choice of reflections for refinement. *R*-factors based on *F*^2^ are statistically about twice as large as those based on *F*, and *R*- factors based on ALL data will be even larger.

### Fractional atomic coordinates and isotropic or equivalent isotropic displacement parameters (Å^2^)

**Table:**

	*x*	*y*	*z*	*U*_iso_*/*U*_eq_	
O1	0.53082 (13)	0.72818 (11)	−0.19967 (6)	0.03281 (19)	
O2	0.68411 (13)	0.78056 (10)	0.11132 (7)	0.03380 (19)	
O3	1.08112 (15)	−0.05781 (10)	0.12226 (7)	0.0384 (2)	
N1	0.71599 (12)	0.47480 (11)	−0.07942 (6)	0.02315 (17)	
N2	0.79478 (12)	0.50033 (10)	0.08008 (6)	0.02230 (17)	
N3	0.90035 (13)	0.22301 (11)	0.03696 (7)	0.02455 (18)	
H3N	0.902 (2)	0.177 (2)	−0.0136 (11)	0.029 (3)*	
C1	0.5650 (2)	0.60945 (19)	−0.26389 (9)	0.0382 (3)	
H1A	0.7060	0.5570	−0.2723	0.057*	
H1B	0.4987	0.6768	−0.3282	0.057*	
H1C	0.5130	0.5152	−0.2340	0.057*	
C2	0.61661 (14)	0.65189 (13)	−0.10610 (8)	0.0241 (2)	
C3	0.59691 (16)	0.76447 (13)	−0.04444 (8)	0.0276 (2)	
H3	0.5249	0.8880	−0.0642	0.033*	
C4	0.69324 (15)	0.67809 (13)	0.04902 (8)	0.02425 (19)	
C5	0.79884 (13)	0.40869 (12)	0.01346 (7)	0.02076 (18)	
C6	0.80040 (19)	0.69559 (16)	0.20397 (9)	0.0340 (2)	
H6A	0.7555	0.6020	0.2436	0.051*	
H6B	0.7846	0.7839	0.2404	0.051*	
H6C	0.9389	0.6440	0.1900	0.051*	
C7	0.99644 (15)	0.09987 (13)	0.12367 (8)	0.0244 (2)	
C8	0.99476 (16)	0.15771 (13)	0.21836 (8)	0.0254 (2)	
H8A	1.0698	0.2384	0.2088	0.030*	
H8B	0.8580	0.2239	0.2317	0.030*	
C9	1.08408 (16)	−0.00098 (13)	0.30737 (8)	0.0268 (2)	
C10	0.96179 (18)	−0.06648 (14)	0.37637 (8)	0.0291 (2)	
H10	0.8237	−0.0125	0.3662	0.035*	
C11	1.0405 (2)	−0.21160 (16)	0.46093 (9)	0.0365 (3)	
C12	1.2469 (2)	−0.28988 (17)	0.47418 (10)	0.0465 (3)	
H12	1.3028	−0.3863	0.5299	0.056*	
C13	1.3710 (2)	−0.22672 (19)	0.40569 (13)	0.0509 (4)	
H13	1.5091	−0.2811	0.4158	0.061*	
C14	1.29121 (19)	−0.08320 (17)	0.32224 (11)	0.0395 (3)	
H14	1.3754	−0.0418	0.2763	0.047*	
C15	0.9033 (3)	−0.2773 (2)	0.53551 (11)	0.0532 (4)	
H15A	0.8517	−0.3510	0.5095	0.080*	
H15B	0.7944	−0.1755	0.5467	0.080*	
H15C	0.9765	−0.3474	0.5978	0.080*	

### Atomic displacement parameters (Å^2^)

**Table:**

	*U*^11^	*U*^22^	*U*^33^	*U*^12^	*U*^13^	*U*^23^
O1	0.0345 (4)	0.0291 (4)	0.0288 (4)	−0.0088 (3)	−0.0081 (3)	0.0008 (3)
O2	0.0394 (4)	0.0197 (3)	0.0398 (5)	−0.0029 (3)	−0.0054 (3)	−0.0126 (3)
O3	0.0558 (5)	0.0174 (3)	0.0345 (4)	−0.0007 (3)	−0.0127 (4)	−0.0084 (3)
N1	0.0225 (4)	0.0204 (4)	0.0253 (4)	−0.0070 (3)	−0.0003 (3)	−0.0044 (3)
N2	0.0225 (4)	0.0166 (3)	0.0269 (4)	−0.0053 (3)	0.0003 (3)	−0.0064 (3)
N3	0.0291 (4)	0.0165 (3)	0.0264 (4)	−0.0042 (3)	−0.0036 (3)	−0.0072 (3)
C1	0.0366 (6)	0.0473 (7)	0.0269 (5)	−0.0117 (5)	−0.0025 (4)	−0.0072 (5)
C2	0.0207 (4)	0.0226 (4)	0.0264 (5)	−0.0082 (3)	−0.0015 (3)	−0.0007 (4)
C3	0.0272 (5)	0.0162 (4)	0.0343 (5)	−0.0042 (3)	−0.0032 (4)	−0.0021 (4)
C4	0.0231 (4)	0.0177 (4)	0.0315 (5)	−0.0058 (3)	0.0008 (4)	−0.0077 (4)
C5	0.0192 (4)	0.0170 (4)	0.0257 (4)	−0.0065 (3)	0.0009 (3)	−0.0049 (3)
C6	0.0396 (6)	0.0300 (5)	0.0348 (6)	−0.0103 (5)	−0.0018 (5)	−0.0148 (4)
C7	0.0264 (5)	0.0181 (4)	0.0277 (5)	−0.0061 (3)	−0.0028 (4)	−0.0061 (3)
C8	0.0285 (5)	0.0194 (4)	0.0254 (5)	−0.0042 (4)	−0.0018 (4)	−0.0066 (3)
C9	0.0311 (5)	0.0195 (4)	0.0272 (5)	−0.0044 (4)	−0.0052 (4)	−0.0067 (4)
C10	0.0358 (5)	0.0253 (5)	0.0268 (5)	−0.0091 (4)	−0.0025 (4)	−0.0092 (4)
C11	0.0591 (8)	0.0274 (5)	0.0258 (5)	−0.0168 (5)	−0.0020 (5)	−0.0084 (4)
C12	0.0640 (9)	0.0279 (6)	0.0379 (7)	−0.0077 (6)	−0.0185 (6)	−0.0002 (5)
C13	0.0398 (7)	0.0373 (7)	0.0596 (9)	0.0001 (5)	−0.0188 (6)	−0.0012 (6)
C14	0.0319 (6)	0.0319 (6)	0.0466 (7)	−0.0050 (5)	−0.0050 (5)	−0.0044 (5)
C15	0.0867 (12)	0.0447 (8)	0.0335 (7)	−0.0317 (8)	0.0095 (7)	−0.0086 (6)

### Geometric parameters (Å, °)

**Table:**

O1—C2	1.3528 (12)	C6—H6C	0.9600
O1—C1	1.4376 (16)	C7—C8	1.5058 (14)
O2—C4	1.3404 (12)	C8—C9	1.5038 (14)
O2—C6	1.4338 (14)	C8—H8A	0.9700
O3—C7	1.2235 (12)	C8—H8B	0.9700
N1—C2	1.3279 (12)	C9—C10	1.3865 (16)
N1—C5	1.3356 (12)	C9—C14	1.3947 (16)
N2—C5	1.3298 (12)	C10—C11	1.3991 (16)
N2—C4	1.3373 (12)	C10—H10	0.9300
N3—C7	1.3734 (13)	C11—C12	1.386 (2)
N3—C5	1.3897 (12)	C11—C15	1.507 (2)
N3—H3N	0.875 (15)	C12—C13	1.384 (2)
C1—H1A	0.9600	C12—H12	0.9300
C1—H1B	0.9600	C13—C14	1.3842 (19)
C1—H1C	0.9600	C13—H13	0.9300
C2—C3	1.3843 (15)	C14—H14	0.9300
C3—C4	1.3839 (15)	C15—H15A	0.9600
C3—H3	0.9300	C15—H15B	0.9600
C6—H6A	0.9600	C15—H15C	0.9600
C6—H6B	0.9600		
			
C2—O1—C1	116.20 (9)	O3—C7—C8	121.00 (9)
C4—O2—C6	117.71 (8)	N3—C7—C8	120.60 (8)
C2—N1—C5	115.15 (8)	C9—C8—C7	111.97 (8)
C5—N2—C4	114.87 (9)	C9—C8—H8A	109.2
C7—N3—C5	132.24 (9)	C7—C8—H8A	109.2
C7—N3—H3N	114.9 (10)	C9—C8—H8B	109.2
C5—N3—H3N	112.9 (10)	C7—C8—H8B	109.2
O1—C1—H1A	109.5	H8A—C8—H8B	107.9
O1—C1—H1B	109.5	C10—C9—C14	119.05 (10)
H1A—C1—H1B	109.5	C10—C9—C8	120.50 (10)
O1—C1—H1C	109.5	C14—C9—C8	120.45 (10)
H1A—C1—H1C	109.5	C9—C10—C11	121.81 (11)
H1B—C1—H1C	109.5	C9—C10—H10	119.1
N1—C2—O1	118.26 (9)	C11—C10—H10	119.1
N1—C2—C3	124.09 (9)	C12—C11—C10	117.89 (12)
O1—C2—C3	117.65 (9)	C12—C11—C15	121.62 (12)
C4—C3—C2	114.49 (9)	C10—C11—C15	120.48 (13)
C4—C3—H3	122.8	C13—C12—C11	121.00 (11)
C2—C3—H3	122.8	C13—C12—H12	119.5
N2—C4—O2	118.59 (9)	C11—C12—H12	119.5
N2—C4—C3	124.04 (9)	C14—C13—C12	120.53 (13)
O2—C4—C3	117.37 (9)	C14—C13—H13	119.7
N2—C5—N1	127.35 (8)	C12—C13—H13	119.7
N2—C5—N3	120.21 (9)	C13—C14—C9	119.71 (13)
N1—C5—N3	112.44 (8)	C13—C14—H14	120.1
O2—C6—H6A	109.5	C9—C14—H14	120.1
O2—C6—H6B	109.5	C11—C15—H15A	109.5
H6A—C6—H6B	109.5	C11—C15—H15B	109.5
O2—C6—H6C	109.5	H15A—C15—H15B	109.5
H6A—C6—H6C	109.5	C11—C15—H15C	109.5
H6B—C6—H6C	109.5	H15A—C15—H15C	109.5
O3—C7—N3	118.39 (9)	H15B—C15—H15C	109.5
			
C5—N1—C2—O1	179.26 (8)	C7—N3—C5—N1	−176.03 (10)
C5—N1—C2—C3	0.03 (14)	C5—N3—C7—O3	−177.41 (11)
C1—O1—C2—N1	−3.14 (14)	C5—N3—C7—C8	3.42 (17)
C1—O1—C2—C3	176.14 (9)	O3—C7—C8—C9	−6.82 (15)
N1—C2—C3—C4	1.02 (15)	N3—C7—C8—C9	172.33 (9)
O1—C2—C3—C4	−178.21 (9)	C7—C8—C9—C10	−101.24 (11)
C5—N2—C4—O2	−179.18 (9)	C7—C8—C9—C14	79.42 (13)
C5—N2—C4—C3	0.40 (14)	C14—C9—C10—C11	0.61 (16)
C6—O2—C4—N2	5.94 (15)	C8—C9—C10—C11	−178.73 (10)
C6—O2—C4—C3	−173.67 (10)	C9—C10—C11—C12	−0.16 (17)
C2—C3—C4—N2	−1.26 (15)	C9—C10—C11—C15	178.87 (11)
C2—C3—C4—O2	178.33 (9)	C10—C11—C12—C13	−0.20 (19)
C4—N2—C5—N1	0.87 (14)	C15—C11—C12—C13	−179.23 (13)
C4—N2—C5—N3	179.79 (9)	C11—C12—C13—C14	0.1 (2)
C2—N1—C5—N2	−1.08 (14)	C12—C13—C14—C9	0.4 (2)
C2—N1—C5—N3	179.93 (8)	C10—C9—C14—C13	−0.70 (19)
C7—N3—C5—N2	4.91 (16)	C8—C9—C14—C13	178.65 (12)

### Hydrogen-bond geometry (Å, °)

**Table:**

*D*—H···*A*	*D*—H	H···*A*	*D*···*A*	*D*—H···*A*
N3—H3N···O3^i^	0.875 (15)	1.979 (15)	2.8535 (12)	176.0 (14)
C3—H3···O2^ii^	0.93	2.52	3.4459 (12)	177.

Symmetry codes: (i) −*x*+2, −*y*, −*z*; (ii) −*x*+1, −*y*+2, −*z*.

## References

[bb1] Allen, F. H., Kennard, O., Watson, D. G., Brammer, L., Orpen, A. G. & Taylor, R. (1987). *J. Chem. Soc. Perkin Trans. 2*, pp. S1–19.

[bb2] Cox, R. A. (1968). *Q. Rev. Chem. Soc.* **22**, 499–526.

[bb3] John, P., Ahmad, W., Khan, I. U., Sharif, S. & Tiekink, E. R. T. (2010). *Acta Cryst.* E**66**, o2048. 10.1107/S1600536810027698 PMC3007500 21588355

[bb4] Mijin, D. & Marinkovic, A. (2006). *Synth. Commun.* **36**, 193–198.

[bb5] Mijin, D. Z., Prascevic, M. & Petrovic, S. D. (2008). *J. Serb. Chem. Soc.* **73**, 945–950.

[bb6] Nogueira, T. C. M., Souza, M. V. N. de, Wardell, J. L., Wardell, S. M. S. V. & Tiekink, E. R. T. (2010). *Acta Cryst.* E**66**, o177. 10.1107/S1600536809053355 PMC2980192 21580062

[bb7] Oxford Diffraction (2010). *CrysAlis PRO* and *CrysAlis RED* Oxford Diffraction Ltd, Yarnton, England.

[bb8] Praveen, A. S., Jasinski, J. P., Golen, J. A., Narayana, B. & Yathirajan, H. S. (2011). *Acta Cryst.* E**67**, o1826. 10.1107/S1600536811024597 PMC3151961 21837194

[bb9] Russell, J. A. (1945). *Annu. Rev. Biochem.* **14**, 309–332.

[bb10] Selig, R., Schollmeyer, D., Albrecht, W. & Laufer, S. (2010). *Acta Cryst.* E**66**, o1132. 10.1107/S1600536810012766 PMC2979114 21579181

[bb11] Sheldrick, G. M. (2008). *Acta Cryst.* A**64**, 112–122. 10.1107/S0108767307043930 18156677

[bb12] Wen, Y.-H., Qin, H.-Q. & Wen, H.-L. (2010). *Acta Cryst.* E**66**, o3294. 10.1107/S1600536810048312 PMC3011411 21589573

[bb13] Wu, W.-N., Cheng, F.-X., Yan, L. & Tang, N. (2008). *J. Coord. Chem.* **61**, 2207– 2215.

[bb14] Wu, W.-N., Wang, Y., Zhang, A.-Y., Zhao, R.-Q. & Wang, Q.-F. (2010). *Acta Cryst.* E**66**, m288. 10.1107/S160053681000471X PMC2983540 21580233

